# Influence of Organic Solvents on Catalytic Behaviors and Cell Morphology of Whole-Cell Biocatalysts for Synthesis of 5′-Arabinocytosine Laurate

**DOI:** 10.1371/journal.pone.0104847

**Published:** 2014-08-19

**Authors:** Meiyan Yang, Hui Wu, Yan Lian, Xiaofeng Li, Furao Lai, Guanglei Zhao

**Affiliations:** 1 State Key Lab of Pulp & Paper Making Engineering, South China University of Technology, Guangzhou, China; 2 College of Light Industry and Food Sciences, South China University of Technology, Guangzhou, China; Queen's University Belfast, United Kingdom

## Abstract

A whole-cell based method was developed for the regioselective synthesis of arabinocytosine laurate. Among the seven kinds of bacteria strains tested in the acylation reaction, *Pseudomonas fluorescens* gave the highest productivity and a higher 5′-regioselectivity than 99%. Compared with pure organic solvents, the use of organic solvent mixtures greatly promoted the yield of the whole-cell catalyzed reaction, but showed little influence on the 5′-regioselectivity. Of all the tested solvent mixtures, the best reaction result was found in isopropyl ether/pyridine followed by isopentanol/pyridine. However, the whole-cells showed much lower thermostability in isopropyl ether/pyridine than in THF-pyridine. To better understand the toxic effects of the organic solvents on *P. fluorescens* whole-cells and growing cells were further examined. Significant influences of organic solvents on the biomass of the cells were found, which differed depending on the type of solvents used. SEM analysis visually revealed the changes in the surface morphology of whole-cells and growing cells cultured in media containing various organic solvents, in terms of surface smoothness, bulges and changed cell sizes. Results demonstrated that organic toxicity to cell structure played an important role in whole-cell mediated catalysis.

## Introduction

Nucleosides and their analogues are very important compounds in medicinal chemistry with antiviral, antitumor and immunosuppressive effects. Arabinocytosine (ara-C) is a non-natural nucleoside with high antileukemic activity [1]. It can be used as a competitor of natural nucleosides, inserted into a growing RNA/DNA strand by the polymerases after phosphorylation and thus disturbing the normal RNA/DNA replication [2]. However, some nucleoside analogues with high hydrophilicity have major clinical shortcomings in the treatment of solid tumors, which might experience a rapid enzymatic deactivation in plasma since they can not transfer across the cell membrane easily due to their high polarity. To improve the clinical efficiency of those analogues, lipophilic modification has gained much attention as a promising strategy, which is also valuable for new nucleoside drug/prodrug discovery and development [3].

The sugar moiety of nucleosides usually contains several hydroxyl groups with similar chemical activities, which makes regioselective acylation of a special hydroxyl groups in nucleoside molecule very difficult via traditional chemical methods [4]. Recently, enzymatic routes especially using lipases and proteinases have become more attractive in modification of nucleoside compounds, and are considered as effective ways to overcome the drawbacks involved in the chemical process [5], [6]. However, the laborious and time-consuming preparation of a pure enzyme (fermentation, separation, purification and sometimes immobilization), as well as their high cost, presently limit their usefulness. Furthermore, in some cases, the isolation of enzymes from their natural environment (eg. the microbial cells) may lead to partial or even complete loss of their activity.

Utilization of a biocatalyst in the form of whole-cells instead of isolated enzymes is a potential way to reduce the cost of industrial processes, since they could avoid the tedious preparation procedures of the enzymes and maintain the enzyme activity by protecting them inside cells. Several processes have benefit from whole-cell biocatalysis, including biodiesel production [7] and production of other high-value compounds, such as vitamins and polyunsaturated fatty acids [8]. The majority of the research on whole-cell mediated catalysis that involves reverse reactions of hydrolysis adopted organic solvents or organic solvent-containing systems. In this research, organic solvents showed significant influence on both the cell activity and autoecology [8]. Recently we have reported our successful synthesis of short chain nucleoside esters by using microbial cells in organic solvent mixtures [9]. The cells showed high regioselectivity and moderate to good substrate conversion in catalyzing the acylation of nucleosides with short chain vinyl esters. Acylation of nucleosides with longer acyl donors can be used for different purposes, since the long chain esters of nucleosides may have higher bioactivity than those with short chains [10]. However, whole-cell catalyzed synthesis of long chain nucleoside esters in organic solvents still remained unexplored. Since microbial enzymes from different sources may be different in substrate recognition for different chain lengths [11], [12], it became necessary to explore microbial cells with capability to catalyze the formation of long-chain nucleoside esters in order to demonstrate the versatility of whole-cell biocatalysts.

**Figure 1:**
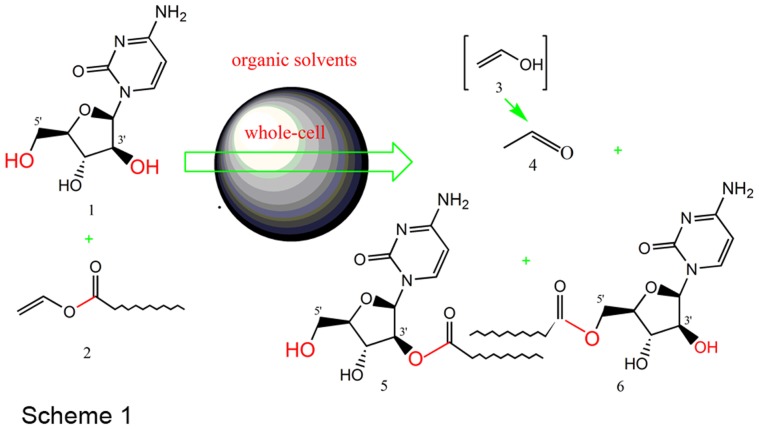
Acylation of ara-C with vinyl laurate catalyzed by whole-cells (1: Ara-C; 2: VL; 3: Unstable enol; 4: Aldehyde; 5: 3′-O-lauryl ara-C; 6: 5′-O-lauryl ara-C).

Encouraged by our recent achievements in whole-cell biotransformation [13], we tried, for the first time, the synthesis of long-chain nucleoside esters in binary organic solvents, using acylation of a well-known antitumor drug ara-C with vinyl laurate as a model reaction (Fig. 1). The influences of different reaction solvents on the biocatalyst activity, product yield and regioselectivity of the reaction were investigated and compared. In particular, in order to make clear the influence of organic solvents on the mechanism of the whole-cell catalyzed reaction, we evaluated the toxic impact of organic solvents on the growth of the whole-cell biocatalysts by biomass and micromorphological analysis.

## Materials and Methods

### 1 Strains and reagents

Pseudomonas aeruginosa GIM1.46, Pseudomonas fluorescens GIM1.209, Pseudomonas stutzeri GIM1.273, Pseudomonas putida GIM1.193, Alcaligenes faecalis GIM1.61, Bacillus subtilis GIM1.135, Bacillus megatherium GIM1.183 were supplied by the Guangdong Institute of Microbiology, Guangzhou (GDIM), China. Arabinocytosine (Ara-C) and Vinyl lurate (VL) were purchased from Sigma (USA). All other chemicals were from commercial sources and were of the highest purity available.

### 2 Cell culture and whole-cell biocatalyst preparation

The precultivation was performed in the medium which contained 1% glucose, 1% beef extract, 1% peptone, 0.5% K_2_HPO_4_ and 0.5% NaCl at 30°C for 24 h. Then 2% seed culture was inoculated to the culture medium containing (g/L) (NH_4_)_2_SO_4_ 5.0, K_2_HPO_4_ 1.0, MgSO_4_·7H_2_O 0.2, soybean oil 5.5 and yeast extracts 1.0. To obtain the whole-cell biocatalyst, the cultivation was carried out in 500 mL flasks containing 100 mL culture media on a rotary shaker at 30°C and 180 rpm. The bacterial cells were harvested by centrifugation to remove the fluid medium, washed twice with distilled water, freeze-dried at −30°C for 24 h and then stored at 4°C.

### 3 Catalysis assays

In a typical reaction, 1 mL organic solvents containing 20 mM ara-C, 900 mM VL, 4% water and 50 mg/mL freeze-dried cells were incubated by shaking (180 rpm) at a fixed temperature for 144 hours. Samples were taken at specified time intervals from the reaction mixture, and then diluted 100 times with pure methanol prior to HPLC analysis. To structurally characterize the product of the reaction, the volumes of components of the reaction were scaled up. Upon completion of the reaction, the reaction mixture was centrifuged to remove the cell masses and then isolated and purified by half-preparation HPLC with a semi-preparative HPLC column. The acquisition was concentrated to about 1 mL by vacuum rotary evaporation. After crystallization under 4°C, two products were obtained as a white powder. All reported data are averages of experiments performed at least in duplicate.

### 4 Bacteria growth in the presence of organic solvents

Bacteria growth in the presence of a second phase was determined based on a method described previously [14]. 2 mL seed culture of *P. fluorescens* after 24 h shaking was transferred to new culture bottles (100 ml) and 1% (v/v) of organic solvent was added. The culture bottles were closed with teflon valves to prevent evaporation and were then incubated for 48 h at 30°C. Then, bacterial growth was evaluated by direct measurement of optical density (OD_560_) using a pure culture fluid as a blank control [15].

### 5 Determination of Protein

The protein was detected with a Bradford Protein Assay KitSuper. After the finished reaction, the whole-cells were removed by syringe filters with 0.45 µm membrane and diluted with PBS to 1 mL, 0.1 mL sample was added to 1 mL Bradford Protein Assay Reagent,mixed sufficiently, reacted for 5 minutes and the optical density detected at 595 nm. All reported data were averages of experiments performed at least in duplicate.

### 6 Solvent sensitivity of whole-cells in organic solvents

The whole-cells were kept in various pure organic solvents under 30°C for 6 h. After the solvents were filtered out, the cells were washed twice using corresponding pure fresh solvents and then added into new co-organic solvent systems to initiate the typical acylation reaction under 30°C for 144 h. Relative activity was estimated as the ratio of product yield after incubated for 6 h to the product yield of non-incubation reactions in corresponding solvent systems.

### 7 SEM analysis

Bacteria was cultured in the media in the presence of 1% organic solvent, based on a method described previously [14]. After that, bacterial mass was centrifuged, freeze-dried and sputter-coated with a thin layer of Au. Then the coated cell samples were analyzed using a Zeiss EVO 18 scanning electron microscope (Germany) with 10 kV accelerating voltage in secondary electron mode. The magnification was 10 K in SEM images.

### 8 HPLC analysis

The reaction mixture was analyzed by RP-HPLC on a 4.6 mm×250 mm (5 µm) Zorbax SB-C18 column (Agilent Technologies Co. Ltd, Massachusetts, USA) with Waters 600E pump and Waters 2996 UV/photodiode array detector (Waters Corp., Massachusetts, USA) at 276 nm. A mixture of ammonium acetate buffer (0.01 M, pH 4.27) and methanol (88/12, v/v) was used as mobile phase at the flow rate of 0.9 mL/min. The retention times for ara-C, 3′-O-laurylara-C and 5′-O-lauryl ara-C were 2.79, 6.92 and 8.03 min, respectively. Regioselectivity was defined as the ratio of the indicated product's HPLC peak area to that of all the products formed upon a certain reaction time. The 5′-O-ester yield (Y) of the reaction was defined as [the moles of 5′-regioisomer in the acylation reaction] ×100%/(initial moles of ara-C). The average error for this determination was less than 1.0%.

### 9 Structure determination

The position of acylation in the prepared ester was determined by ^13^C NMR (Bruker AVANCE Digital 400 MHz Nuclear Magnetic Resonance Spectrometer, Bruker Co., Germany) at 100 MHz. DMSO-*d*_6_ was used as solvent and chemical shifts were expressed in ppm shift. The LC-MS spectra of the product were recorded on a Agilent 1290/Bruker MaXis Impact Plus ESI Mass Spectrometer with a spray voltage of 4.5 kV (Bruker Co., Germany).

#### Ara-C

^13^C-NMR δ 164.9 (C-4), 154.3 (C-2), 143.4 (C-6), 92.5 (C-5), 86.5 (C-1′), 85.3 (C-4′), 76.3 (C-3′),74.9 (C-2′), 61.2 (C-5′); FT-IR (KBr, cm-1) v 3214-3342 (OH,NH), 2922 (CH), 1649 (C = C), 1117/1051 (C-O-C).

#### 5′-O-lauryl ara-C

^13^C-NMR(DMSO-*d*_6_, 100 MH_Z_) δ: 173.29 (COO), 165.80 (C4-N), 155.62 (C = O 2), 143.37(C-6), 93.35(C-5), 86.97(C-1′), 82.55(C-4′), 77. 27 (C-3′), 75.09 (C-2′), 64.16 (C-5′), 22.52-34.04 (CH_2_), 13.81 (CH_3_). ESI-MS ((C_21_H_35_N_3_O_6_, 425.53): m/z = 424.20(100) ([M-H]^+^).

#### 3′-O-lauryl ara-C

^13^C-NMR(DMSO-*d*_6_, 100 MH_Z_) δ: 172.36 (COO), 164.45 (C4-N), 155.85(C = O 2), 144.46 (C-6), 93.49 (C-5), 86.90 (C-1′), 83.02 (C-4′), 79. 16 (C-3′), 72.95 (C-2′), 61.42(C-5′), 25.65-34.27(CH_2_), 14.48 (CH_3_);ESI-MS (C_21_H_35_N_3_O_6_, 425.53): m/z = 424.20 ([M-H]^+^).

## Results

### 1 Screening of strains

It is well-known that enzymes of various sources may have different substrate specificities. To screen highly efficient microbial biocatalysts for acylation of ara-C with VL, seven strains from 3 bacterial genera were examined for their catalytic behavior using traditional organic solvent as reaction media (Table 1). Unexpectedly, only *P. fluorescens* GIM1.209 showed catalytic activity with higher 5′-regioselectivity than 99% among four *Pseudomonas* strains tested. No 2′-ester products were detected. Another strain of *Bacillus megatherium* also can catalyze the acylation reaction but gave much lower initial rate and product yield than *P. fluorescens* GIM1.209. Thus, *P. fluorescens* GIM1.209 was chosen as the best biocatalyst for further research.

**Table 1:** Acylation of ara-C with vinyl laurate by various bacteria^a^.

Strains^b^	V_0_ (mmol/L·h)	Y (%)	5′-regioselectivity (%)
*Pseudomonas aeruginosa* GIM1.46	ND^c^	ND	ND
*Pseudomonas fluorescens* GIM1.209	1.19±0.06	10.11±0.08	>99
*Pseudomonas stutzeri* GIM1.273	ND	ND	ND
*Pseudomonas putida* GIM1.193	ND	ND	ND
*Alcaligenes faecalis* GIM 1.61	ND	ND	ND
*Bacillus subtilis* GIM1.135	ND	ND	ND
*Bacillus megatherium* GIM 1.183	0.08±0.01	3.27±0.02	>99

a Reaction condition: 50 mg/mL whole cells, 20 mmol/L ara-C, 500 mmol/L VL, 40 µL water, 1 mL 25% (v/v) IPE-pyridine, 30°C,180 rpm. The data were obtained after 144 h reaction.

b The bacteria were cultured at 30°C and 180 rpm for 48 h and then the harvested cells were pretreated by lyophilization for 24 h.

c Not detected.

### 2 Effect of organic solvents on synthesis of ara-C laurate catalyzed by *P. fluorescens*

Due to the high polarity of the nucleoside substrate, three traditional high-polar solvents were chosen as reaction media to form a homogeneous solution. However, no reaction occurred when the lyophilized *P. fluorescens* cells were added in these pure solvents containing two substrates (Table 2, entries 1–3). Thus, a “co-solvent” strategy was applied using the mixture of a polar solvent (pyridine) and a hydrophobic solvent as the reaction system. If the presence of hydrophobic co-solvents could aid in preventing the inactivation of the biocatalyst caused by the polar solvent, it is thus important to explore the catalytic behaviors of the whole-cell biocatalysts in such binary solvent systems. The results in Table 2 (entries 4–8) clearly showed that the use of binary solvent mixture had significant influence on the whole-cell catalyzed acylation. The cells showed much higher regioselectivity than 99% in the organic solvents tested. In all the five kinds of binary solvent mixtures designed, both the initial rates and product yields of the reaction were disappointedly low.

**Table 2:** Acylation of ara-C with vinyl laurate by *P. fluorescens* in different organic solvent systems^a^.

Entry	Organic solvent^b^	V_0_ (mmol/L·h)	Y (%)	5′-regioselectivity (%)
1	Pyridine	0	0	0
2	DMF	0	0	0
3	DMSO	0	0	0
4	Acetonitrile/pyridine	2.70±0.13	6.82±0.13	>99
5	Isopentanol/pyridine	3.18±0.15	9.23±0.16	>99
6	THF/pyridine	2.42±0.12	4.58±0.12	>99
7	IPE/pyridine	1.19±0.06	10.11±0.08	>99
8	Hexane/pyridine	1.69±0.04	4.05±0.04	>99

a The reaction conditions: 50 mg/mL biocatalyst, 20 mmol/L ara-C, 500 mmol/L VL, 40 µL water, 1 mL organic solvents, 30°C, 180 rpm and reaction time of 144 h.

b The volume ratio of two organic solvent used, 1∶3 (v/v).

### 3 Solvent sensitivity of the whole-cells

**Figure 2:**
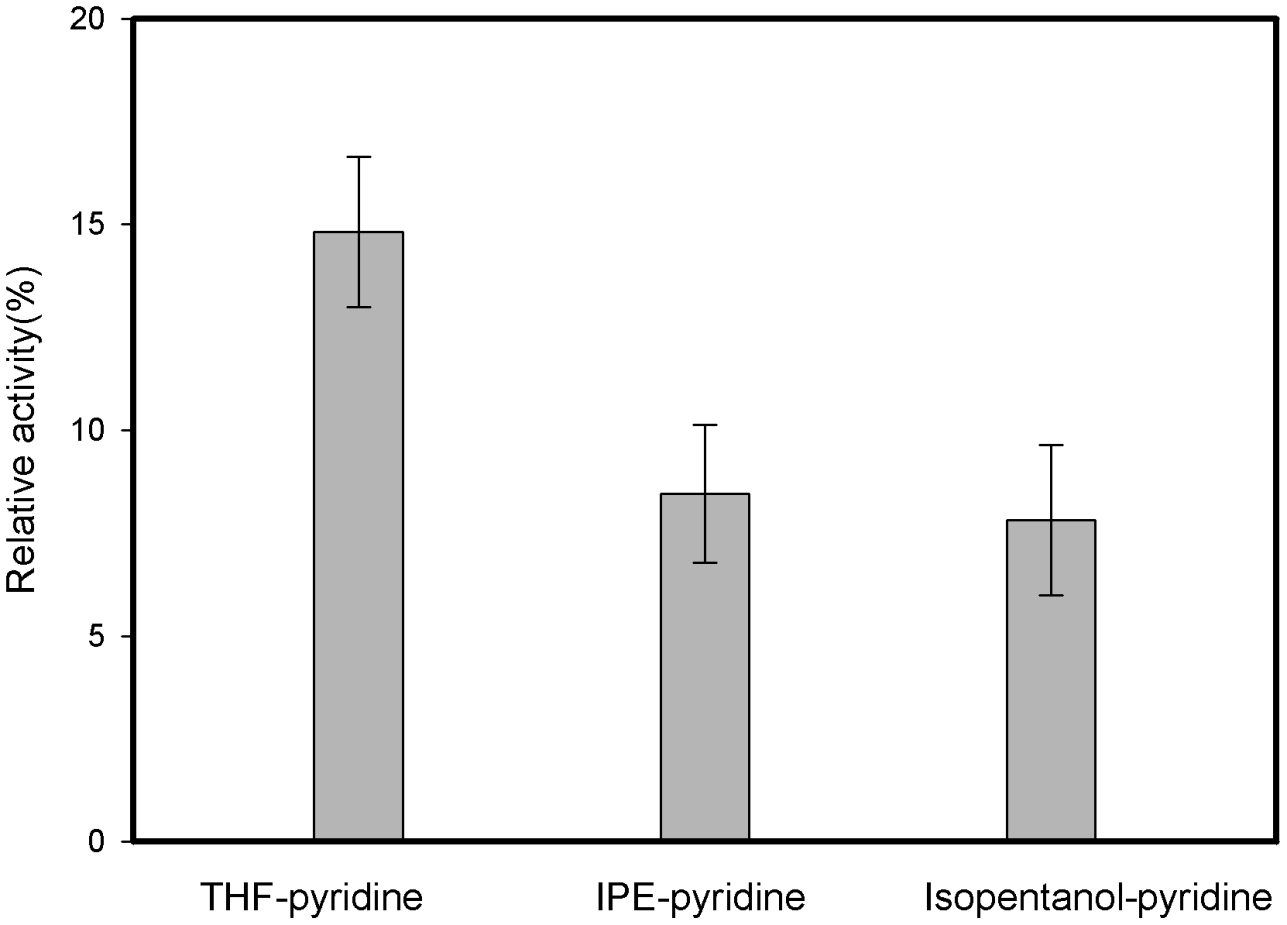
Solvent-sensitivity of the whole-cells in organic solvents (The whole-cells were kept in different mixture organic solvents under 30°C for 6 h before catalyzed the reaction).

To make clear the solvent sensitivity of the whole-cell biocatalysts, the relative catalytic activities of the heat-treated whole-cells in different binary organic solvents were compared. As shown in Fig. 2, after a 6-hour incubation in various organic solvents at 30°C, the whole-cell catalysts in all the tested co-solvents showed an evident decrease in the relative activity to less than 15%.

### 4 Effects of different organic solvents on surface morphology of the whole-cells

Lyophilized cells were incubated in different organic solvents and then their surface morphologies were analyzed by SEM to gain insight into the influence of organic solvents on the whole-cell biocatalysts.

**Figure 3:**
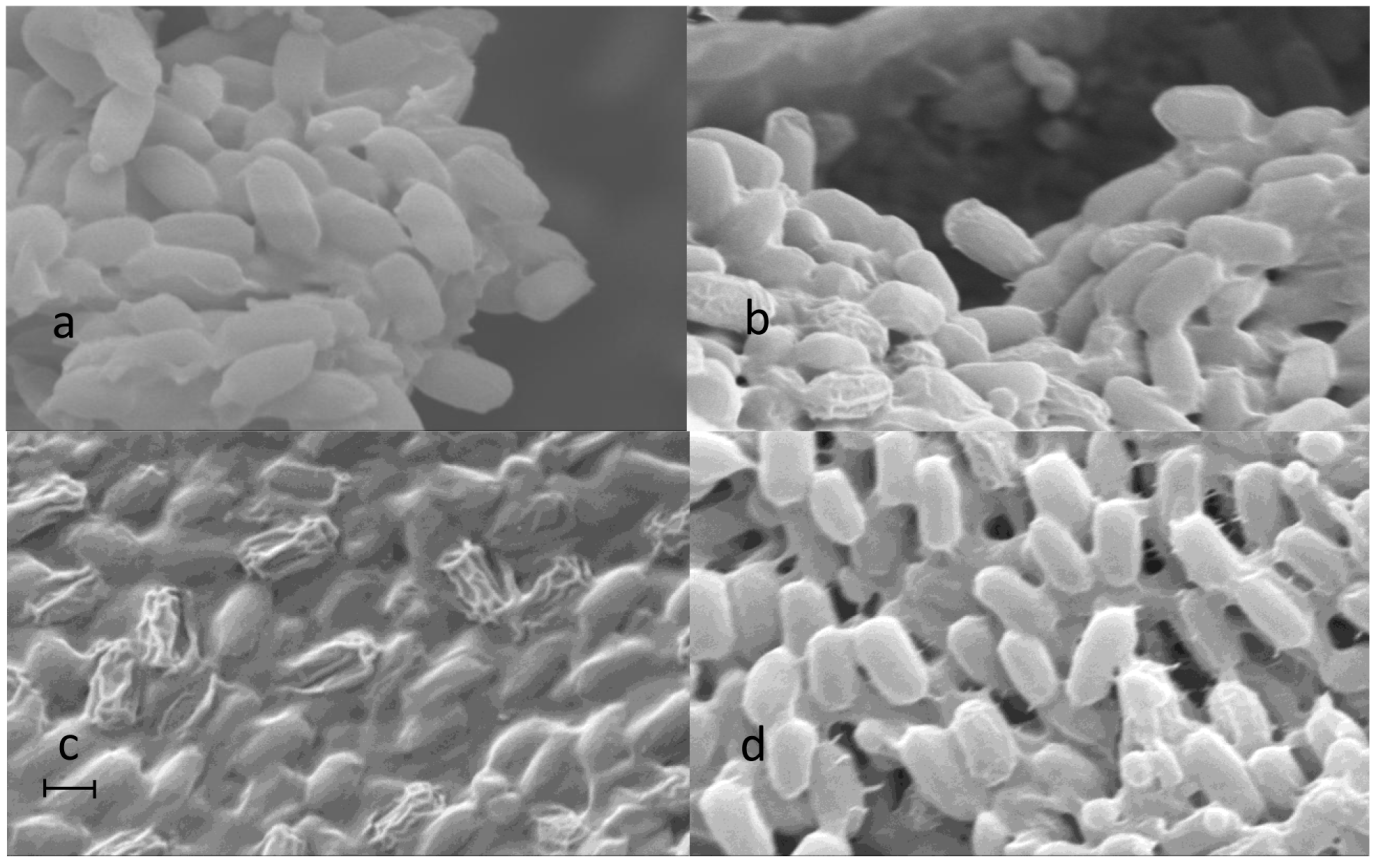
SEM photographs of freeze-dried *P. fluorescens* cells after incubated in organic solvents for 24 h {a: PBS, b: DMF, c: IPE/pyridine (v/v = 1∶3), d: THF/pyridine (v/v = 1∶3)}, magnification was 10 K, scale bar: 1 µm.

Fig. 3 illustrates the surface morphologies of whole-cells incubated in PBS buffer solution (control) and different kinds of pure and mixed organic solvent systems. After incubated in PBS buffer solution for 24 h, the cells showed the normal shapes of P. fluorescens with smooth surfaces (Fig. 3a). When incubated with three pure organic solvents, evident wrinkling of the cells such as Fig. 3b (DMF) was found in other tested solvents (Fig. S1. C–D).

When incubated with different co-organic solvents which were used for catalysis reactions previously (pyridine content 75%, v/v), more serious wrinkling phenomena of the whole-cell envelope than that observed for pure organic solvents was observed in the treatments containing acetonitrile, isopentanol, IPE and n-hexane (Fig. S1. E–G), some wrinkling cells even shriveled and lost their normal bacilliform (Fig. 3c). Compared to other co-solvents, the cells in THF/pyridine (Fig. 3d) showed fewest and slightest wrinkling, in which cells always kept whole bacilliform despite wrinkling.

### 5 Toxic effect of various organic solvents on biomass of *P. fluorescens*

For a deep understanding of the effects of organic solvents on whole-cell catalyzed acylation of nucleosides, we initially explored the toxic effect of various organic solvents on whole-cell biocatalysts by a comparative study of the biomass of *P. fluorescens* in various organic solvents, in terms of the OD_560_ values. As shown in Table 3, the addition of organic solvents greatly inhibited the growth of *P. fluorescens* cells. In tetrahydrofuran (THF) and isopropyl ether (IPE), higher OD_560_ values than those in other solvents were found.

**Table 3:** Effects of organic solvents on cell growth of *P. fluorescens*.

Solvents^a^	lg*P* b	OD_560_	Solvents^a^	lg*P* b	OD_560_
Purified water	—	2.26±0.05			
DMSO	−1.30	1.45±0.11	Pyridine	0.71	0.18±0.15
Dimethylformamide	−1.00	1.38±0.07	IPE	1.90	2.00±0.09
THF	0.49	1.99±0.06	n-Hexane	3.50	1.63±0.12

a Organic solvent content 1% (v/v). OD_560_ values given here show the increase in OD_560_ of the medium from 0 h to 48 h, and thus indicate the biomass cultured in media containing various organic solvents.

b lg*P* value of the organic solvents used was from Ref. (Trends in Biotechnology, 1995, 13(2):63–70) (v/v).

### 6 Morphology changes of the cells grown in organic solvent-containing systems

**Figure 4:**
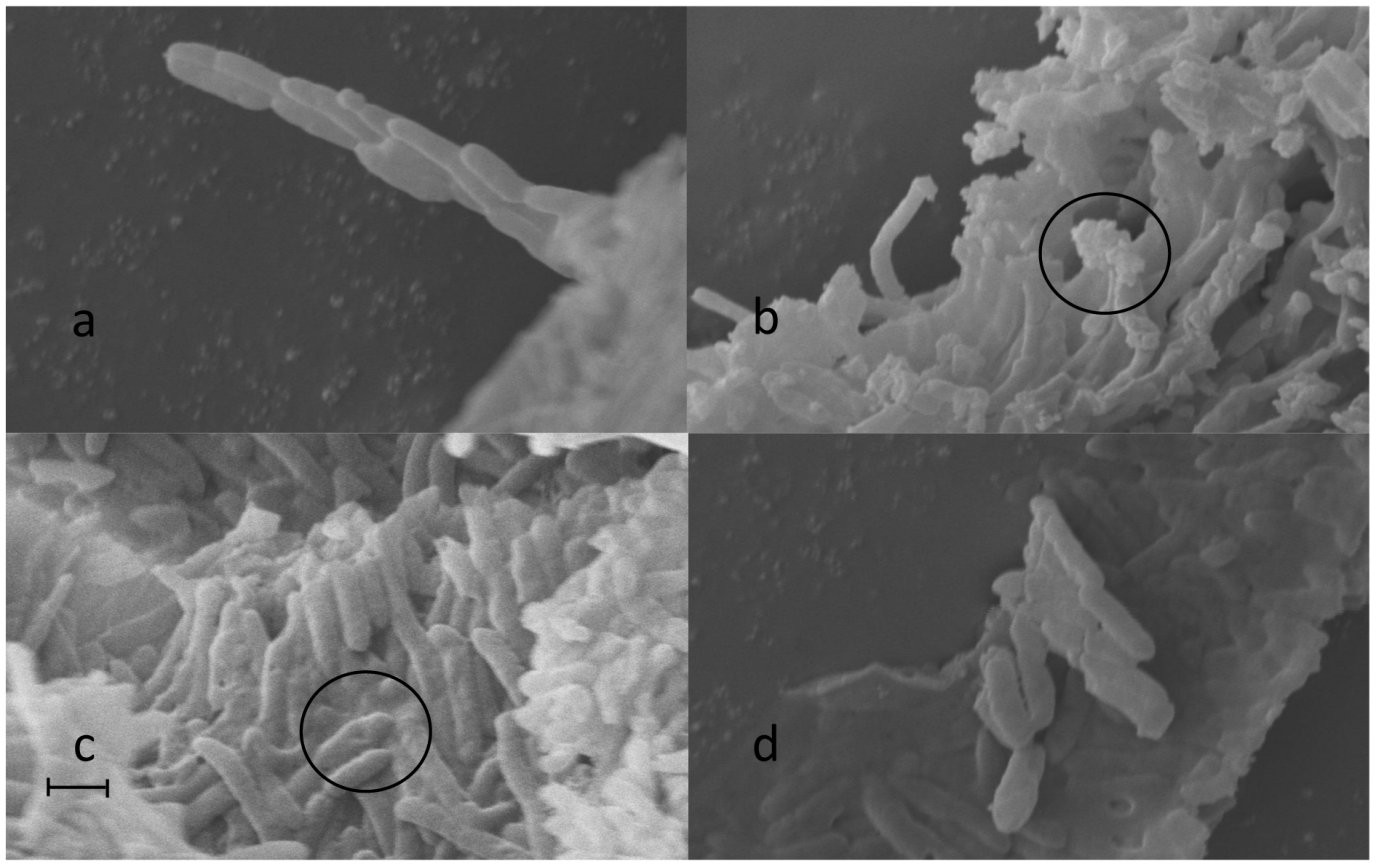
SEM photographs of freeze-dried *P. fluorescens* grown in the presence of 1% different organic solvents (a: water, b: Pyridine, c: DMSO, d: IPE), magnification was 10 K. Circles: b–cotton-like debris, c–sags, scale bar: 1 µm.

From a theoretical viewpoint, it was of considerable importance to investigate the influence of solvent mixtures on cell structure, especially the surface morphology of the bacterial biocatalyst. This effort may also be helpful in explaining the toxic effect of organic solvent on cells. By using SEM, the micromorphologies of *P. fluorescens* cultivated with media containing different organic solvents were examined. Fig. 4a shows the SEM photograph of *P. fluorescens* cells in their normal state. The cells are straight bacilliform with an average size of 2.28 µm×0.50 µm, and arranged orderly by connecting head to tail one by one. Fig. 4b–d shows the same cells in the organic solvent-affected states. When cultivated with organic solvents tested, all the cells lyophilized under the same conditions arranged disorderly. In the medium containing DMSO with the lowest lg*P* value (−1.30), the cells showed a rough surface with small pits which, however, also were found in cells in IPE with much higher lg*P* value (Fig. 4c, d). Obvious surface bulges were found when *P. fluorescens* cells were grown in a medium containing pyridine (lg*P*, 0.71). Cells showed curved shapes with a lot of cotton-like debris on both ends of the cell (Fig. 4b). Hydrophobic solvent n-hexane (lg*P*, 4.7) showed little effect on the surface morphology of the cells. The solvents tested also affected cell sizes.

## Discussion

This paper firstly investigated the catalytic behaviors of microbial whole-cells in acylation of a long-chain nucleoside ester in organic solvent systems. The results of strain screening confirmed that the whole-cell biocatalysts from different sources have different catalytic efficiency towards the synthesis reaction of long-chain nucleoside ester, which may be because the cell-bound lipases responsible for the acylation varied with the strains of the whole cells tested. The strain, *P. fluorescens* GIM1.209, was successfully screened out as the whole-cell biocatalyst used in the acylation of ara-C luarate. Although the initial reaction rate and product yield of the whole-cell catalyzed acylation were somewhat low, much higher 5′-regioselectivity than 99% was achieved. This could be an advantage over chemical procedures for regioselective synthesis of nucleoside esters, since the chemo-process requires tedious steps and severe reaction conditions to proceed.

Organic solvents may offer attractive changes in the selectivity of the reaction and suppression of water-dependent side reactions [16]. However, there was a limitation of solvent adoption previously reported by our group that polar solvents were required to dissolve the polar substrate but also resulted in deactivation of the biocatalyst [17]. Considering the solubility of ara-C, the catalyzing reaction of ara-C with VL was conducted in pure DMSO, DMF and pyridine but no reaction occurred, which might be because the related lipase naturally located on the cell surface may be inactivated by the polar solvents. Then a “co-solvent” strategy was applied. The use of binary solvent mixtures significantly improved the catalytic activity of the whole-cells in the reaction, though both the initial rates and product yields of the reaction were still low. No obvious correlation was found between the catalytic activity of the biocatalysts and the polarity of the solvent mixture. The low catalytic activity of the biocatalyst observed was partially due to the water-stripping capability of organic solvents from the molecular surface of the enzymes bound on the cells. In addition, organic solvents may have different toxic effects on the microbial cells, which may also account for the poor catalytic activity of the whole-cells.

The incubation pre-treatment of *P. fluorescens* cells in different organic solvents at a mild temperature (30°C) greatly reduced their catalytic activity in the subsequent acylation reaction, strongly indicating that *P. fluorescens* cells were rather sensitive to organic solvents. Considering the catalytic behaviours of the whole-cells in different solvents, it was therefore possible that organic solvents had a deactivation effect on cell-bound lipases for the long-chain ester synthesis reaction via enzyme-solvent interactions [18], [19]. In addition, the organic solvents may also have a destructively toxic effect on the cell morphology of the whole-cell biocatalysts. Hence, organic solvents may affect the catalytic performance of whole-cell biocatalysts in different ways from their effects on previously-reported isolated enzymes [20]. The observed relatively lower residual activity of the cells in an IPE-containing system than THF-containing system may thus be partially due to the less toxic effects of THF than IPE on the cells of *P. fluorescens*.

It is well-known that organic solvents, commonly-used for nonaqueous biocatalysis, are toxic to isolated enzymes. For whole-cell mediated reactions, organic solvents might affect not only the structures of enzymes bound in cells, but also the cell structures [21]. However, the visual changes of the cell surface remain poorly studied. In order to find out the mechanism of toxic effects on whole-cell biocatalysts, SEM analysis was done to further investigate the changes of cell structure. The SEM photographs showed that the whole-cells envelope underwent serious wrinkling phenomena in most treatments, but it was relatively slight in THF/pyridine. It was well considered that the activity of enzymes was higher in hydrophobic than hydrophilic solvents, thus the observed loss in total catalytic activity of whole-cell biocatalyst in the acylation performed in THF/pyridine may be mainly due to the medium polarity of THF. Although more evident wrinkling phenomena of the cell envelope was observed in IPE/pyridine, it gave the highest productivity of the whole-cells among all the solvents tested. So when incubated in mixed organic solvents, the extent and the quality of wrinkled cells varied with the different co-solvents used.

The interesting wrinkling of the wall envelope of whole-cells when incubated with organic solvents clearly showed that the organic solvents had toxic effects on the whole-cell biocatalyst of P. fluorescens. It has been widely accepted that the wrinkling of the wall envelope of bacterium is forced by dehydration of the cytoplasm [22], which however was not suitable for explaining the wrinkling phenomenon presented here since lyophilized cells were used as biocatalysts. As a typical strain of Gram-negative bacteria, P. fluorescens has a thinner peptidoglycan layer and an additional outer membrane. Both the membranes mainly consist of a lipid bilayer in which a large number of enzymes and transport proteins may be embedded [7], [23], [24]. The organic solvents with strong polarities wrapped the cells, interacted with the outer membrane and then the plasma membrane, changing the surface charge and hydrophobicity of the cells [21], when the changes reached a limitation, the membrane broken. And therefore, some cellular contents of the cells may be leaked during the treatment before SEM analysis. From the point of view of whole-cell biocatalysis, the partial disruption and increased permeability of the cell wall and plasma membrane may reduce the inner mass-transfer limitation of the substrates into the enzyme molecules bonded on the cell membrane and located inner cells, and thus improve reaction efficiency. But the strong polarities of organic solvents could break the higher molecular structure and consequently deactivated the enzymes, so the more the enzymes are exposed to the organic solvents, the lower whole-cell activity was. This explains why the productivities presented were a little low when catalyzing the synthesis of ara-C luarate by P. fluorescens whole-cells in organic solvents. Bradford Protein Assay KitSuper showed detectable protein contents in organic reaction mixtures after separation of the cells from the media, which confirmed our conclusion of influence of organic solvents on cell permeability deduced based on the SEM data.

Since the toxicity of organic solvents towards enzymes can be estimated by the lgP [19], we also tried here to correlate the toxic effect of organic solvents on microbes with their lgP values. However, no trend of organic solvent toxicity to the cell surface was found with increasing lgP value of the organic solvents. The SEM results demonstrated that some of the toxicological effects may not be entirely due to their lgP value, since each organic solvent has its own specific properties and consequently will affect organisms in different ways.

Another interesting result of SEM observation was the morphology changes of the cells grown in organic solvent-containing systems. Although several previous researches revealed the relationship between the lg*P* values and the toxicity of given organic solvents [23], [25], no significant evidence for such a relationship was found in cultivation of *P. fluorescens* cells. The results demonstrated that organic solvent could significantly change the morphology of the cells, even disrupt the bacterial cells. Besides, the toxicity to *P. fluorescens* cells greatly differed with the type of solvent used. In the mixture of THF and IPE, higher OD_560_ values of the culture than those in other solvents indicated that THF and IPE might be less toxic to the growth of *P. fluorescens* cells. The most probable sites that organic solvents acted on are the phospholipids outer membrane (part of the bacterial cell wall) and plasma membrane of gram-negative bacteria. Both the membranes mainly consist of a lipid bilayer in which a large number of enzymes and transport proteins may be embedded [11], [12], [24]. The influence caused by organic solvents may thus affect the structural and functional integrity of the cell [26], [27]. From the point of view of whole-cell biocatalysis, the organic solvent toxicity to cell structure may bring double effects on the catalytic behavior of the whole-cell biocatalyst. One is that partial disruption of the cell wall and membrane by organic solvents may result in lower stability and more loss of the cell-bound enzyme, thus reducing the catalytic efficiency of the biocatalyst. The other is that the disruption of the cell wall and plasma membrane may reduce the inner mass-transfer limitation of the substrates into the enzyme molecules bonded on the cell membrane, and at the same time, the enzyme was exposed to and inactivated by the high-polar organic solvents. Therefore, the observed different catalytic behaviors and sensitivity of whole-cells to various organic solvents were a result of a comprehensive effect of the organic solvents in interrupting the cell structure and reducing the cell-bond enzyme stability.

## Conclusions

This research firstly investigated the catalytic behavior of the microbial whole-cells in acylation of long-chain nucleoside esters in organic solvent systems. Although the initial reaction rate and product yield of whole-cell catalyzed acylation were still low, much higher 5′-regioselectivity than 99% was found. This new whole-cell route in organic solvents could be an advantage over chemical procedures for regioselective synthesis of nucleoside esters, since the chemoprocess requires tedious steps and severe reaction conditions to proceed. The study also demonstrated the toxicity effect of organic solvents on the whole-cell biocatalyst from *P. fluorescens*, which provides knowledge required for the wider use and modification of strains with organic solvent tolerance for biotechnological applications. However, challenges still remain to be conquered for the practical application of this whole-cell biocatalytic route, especially the improvement of reaction efficiency.

## Supporting Information

Figure S1 SEM photographs of freeze-dried *P. fluorescens* cells. **A–B**: Cells grown in the presence of 1% different organic solvents for 48 h (A: THF, B: n-Hexane); **C–G**: Cells after incubated in organic solvents for 24 h {C: DMSO, D: pyridine E: Acetonitrile/pyridine (v/v = 1∶3), F: Isopentanol/pyridine (v/v = 1∶3), G: n-Hexane/pyridine (v/v = 1∶3)}, magnification was 10 K, scale bar: 1 µm.
(TIF)
