# Supplementary figures and images for: Influence of Organic Solvents on Catalytic Behaviors and Cell Morphology of Whole-Cell Biocatalysts for Synthesis of 5′-Arabinocytosine Laurate
Source: PLoS One. 2014 Aug 19;9(8):e104847. doi: 10.1371/journal.pone.0104847 (PMC4138074; doi:10.1371/journal.pone.0104847)

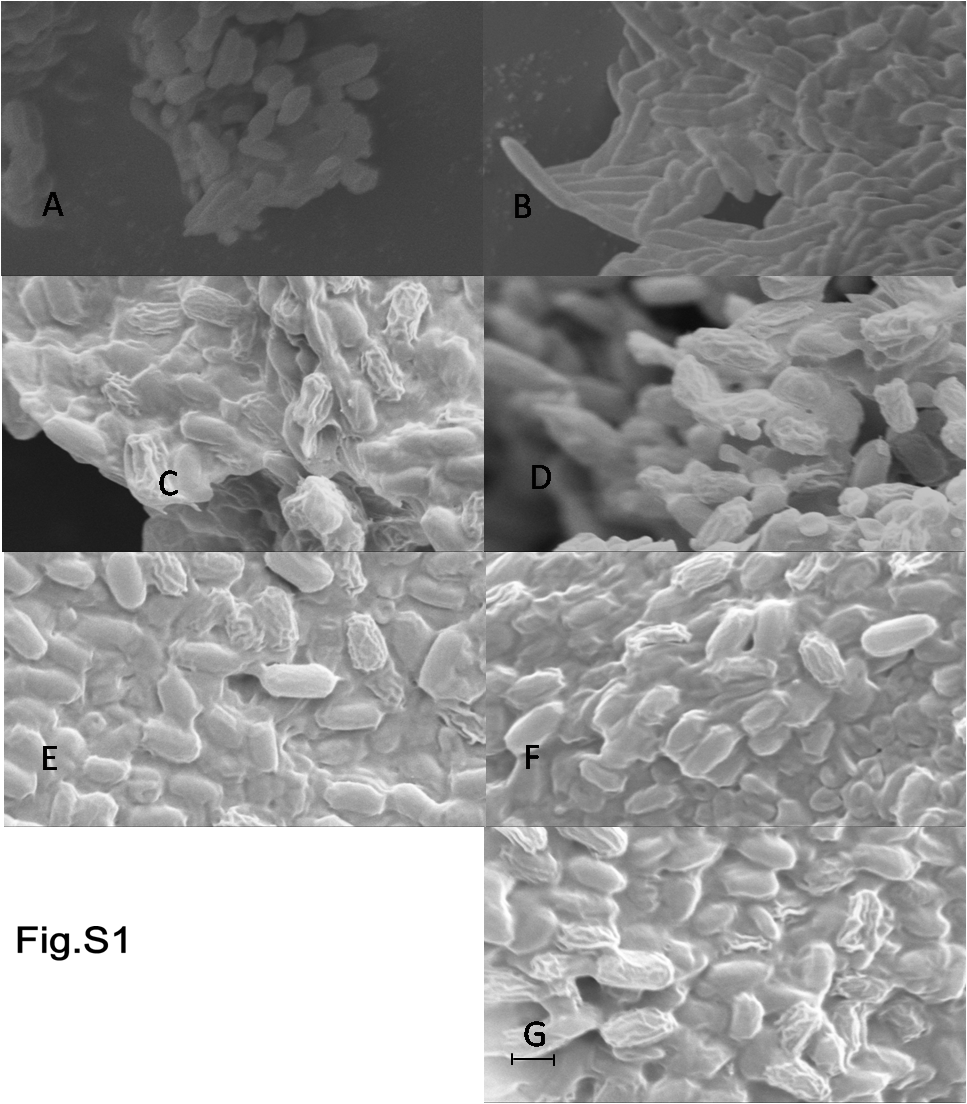

Supplement: Figure S1 — SEM photographs of freeze-dried P. fluorescens cells. A–B: Cells grown in the presence of 1% different organic solvents for 48 h (A: THF, B: n-Hexane); C–G: Cells after incubated in organic solvents for 24 h {C: DMSO, D: pyridine E: Acetonitrile/pyridine (v/v = 1∶3), F: Isopentanol/pyridine (v/v = 1∶3), G: n-Hexane/pyridine (v/v = 1∶3)}, magnification was 10 K, scale bar: 1 µm. (TIF) [file pone.0104847.s001.tif]
